# Serum-Based Proteomic Approach to Identify Clinical Biomarkers of Radiation Exposure

**DOI:** 10.3390/cancers17061010

**Published:** 2025-03-17

**Authors:** Emeshaw Damtew Zebene, Biagio Pucci, Rita Lombardi, Hagos Tesfay Medhin, Edom Seife, Elena Di Gennaro, Alfredo Budillon, Gurja Belay Woldemichael

**Affiliations:** 1 Nuclear Medicine Unit, Department of Internal Medicine, College of Health Sciences, Addis Ababa University, Addis Ababa 9086, Ethiopia; emeshaw.damtew@aau.edu.et (E.D.Z.); hagos.tesfay@aau.edu.et (H.T.M.); 2 Department of Microbial Cellular and Molecular Biology, College of Natural and Computational Sciences, Addis Ababa University, Addis Ababa 9086, Ethiopia; gurja.belay@aau.edu.et; 3 Experimental Pharmacology Unit, Laboratory of Naples and Mercogliano (AV), Istituto Nazionale Tumori—IRCCS—Fondazione G. Pascale, 80131 Naples, Italy; b.pucci@istitutotumori.na.it (B.P.); e.digennaro@istitutotumori.na.it (E.D.G.); 4 Experimental Animal Unit, Istituto Nazionale Tumori—IRCCS—Fondazione G. Pascale, 80131 Naples, Italy; 5 Radiotherapy Center, College of Health Sciences, Addis Ababa University, Addis Ababa 9086, Ethiopia; edomseife3@gmail.com; 6 Scientific Directorate, Istituto Nazionale Tumori—IRCCS—Fondazione G. Pascale, 80131 Naples, Italy; a.budillon@istitutotumori.na.it

**Keywords:** biomarker, head and neck cancer, ionizing radiation, radiotherapy, serum proteomics

## Abstract

Background: Ionizing radiation (IR) exposure poses a significant health risk due to its widespread use in medical diagnostics and therapeutic applications, necessitating rapid and effective biomarkers for assessment. Objective: The aim of this study is to identify the serum proteomic signature of IR exposure in patients undergoing radiotherapy (RT). Methods: Blood samples were obtained from eighteen patients with head and neck cancer (HNC) and five patients with rectal cancer before and immediately after they underwent curative intensity-modulated radiotherapy (IMRT). The comprehensive serum proteome was analyzed in individual samples using nanoHPLC-MS/MS. Results: Forty radiation-modulated proteins (RMPs), 24 upregulated and 16 downregulated, with a fold change ≥1.5 and *p*-value < 0.05 were identified. About 40% of the RMPs are involved in acute phase response, DNA repair, and inflammation; the key RMPs were *ADCY1*, *HGF*, *MCEMP1*, *CHD4*, *RECQL5*, *MSH6*, and *ZNF224.* Conclusions: This study identifies a panel of serum proteins that may reflect the radiation response, providing a valuable molecular fingerprint of IR exposure and paving the way for the development of sensitive and specific biomarkers for early detection and clinical management of IR-related injuries.

## Simple Summary

Ionizing radiation (IR) is commonly used in medical diagnostics and therapeutic applications, such as radiotherapy (RT) for cancer treatment. IR can damage DNA, potentially leading to mutations that may result in cancer over time. High doses of IR can cause immediate health effects known as acute radiation syndrome. Identifying effective biomarkers to monitor and assess the impact of IR exposure is critically important. This study reports a panel of potential protein biomarkers, focusing on those involved in the acute phase response, DNA repair, and inflammation in patients undergoing definitive radiotherapy for cancer treatment.

## 1. Introduction

We are exposed to ionizing radiation (IR) in various aspects of life due to its diverse applications in diagnostics, therapeutics, agriculture, and industries. In addition to this, we are vulnerable to cosmic rays and naturally occurring radon [1]. Individuals may be exposed to varying doses and types of ionizing radiation, including X-rays, gamma rays, protons, or heavy ions, during manned space missions, radiation therapies, industrial accidents, or nuclear incidents [2]. Medical therapy and diagnostic procedures increasingly apply ionizing radiation. In 2018, the International Agency for Research on Cancer (IARC) reported 18 million new cancer cases; of these, 50–60% involved radiotherapy [3,4]. For instance, in the USA, image-guided interventions and medical imaging radiation exposure has increased six-fold since 1980 [5].

Exposure to high or lethal doses of radiation can lead to the development of acute radiation syndrome (ARS), characterized by various clinical manifestations. These may include gastrointestinal disorders, retinal or skin lesions, internal bleeding, and, in severe cases, even death. These clinical presentations can serve as diagnostic indicators of underlying radiation-induced damage [6]. In most real-world scenarios, individuals are more commonly exposed to low or non-lethal levels of radiation. In these cases, the resulting damage is often gradual and subtle, manifesting as an increased risk of carcinogenesis. This poses a significant challenge for radiation safety management, as the ability to rapidly and efficiently triage low-dose-exposed individuals within large populations becomes a critical requirement [7].

Biomarkers of human exposure to ionizing radiation (IR) are necessary for evaluating normal tissue damage in radiation oncology, as well as for bio-dosimetry in nuclear accidents and accidental radiation exposures. Physical symptom monitoring, i.e., the onset of nausea and kinetics of circulating blood lymphocytes, and cellular markers, i.e., cytogenetic biodosimetry, which quantifies IR-induced chromosomal damage in circulating blood lymphocytes, are present-day practices in radiation biodosimetry. While these techniques are highly sensitive and specific, they are time-consuming and cannot provide quick results, making it difficult to detect individuals who require immediate medical intervention [8,9].

Given these limitations, there is a need for research of the molecular biomarkers of ionizing radiation injury to develop methods that can provide early and rapid diagnosis of radiation casualties and complement the existing biological dose estimation techniques. Exposure to ionizing radiation has been found to regulate specific biomarkers that influence cancer treatment outcomes [10]. Moreover, the link between radiation and inflammation is crucial, given that inflammation significantly affects the body’s response to radiation [11]. Recent reviews have focused on protein biomarkers in normal tissues related to ionizing radiation and their association with radiosensitivity, underscoring the need to comprehend these biomarkers in relation to radiation exposure [3]. Additionally, biomarkers related to DNA repair and cell cycles have been recognized as important indicators of radiation exposure and inflammatory stress in human blood [12].

Quantitative proteomics has emerged as an effective analytical method for thoroughly examining proteins that are expressed under specific conditions, such as in disease states or in response to external stimuli, like ionizing radiation. Over the past few decades, significant advancements in mass spectrometry technology have dramatically increased the capacity for accurate protein identification and quantification. These technological improvements have propelled proteomics to the forefront of biomedical research, with scientists increasingly leveraging these capabilities to discover novel biomarkers and potential therapeutic targets [13]. Furthermore, to effectively assess radiation exposure levels and the extent of injury, especially in emergencies, it is crucial to integrate various ‘omics’ approaches, such as genomics and proteomics [14].

In this study, we conducted a comprehensive serum proteomics analysis to identify the characteristic profile of the radiation response and to determine the significance of essential molecular and cellular mechanisms that play a role in the reaction to partial body irradiation in cancer therapy.

## 2. Materials and Methods

### 2.1. Patient Characteristics and Sample Collection

This research was conducted at the radiotherapy center of Tikur Anbessa Specialized Hospital, which is part of the College of Health Sciences at Addis Ababa University in Ethiopia. The objective was to identify the serum proteomic signature of ionizing radiation (IR) exposure in patients undergoing definitive radiotherapy (RT). The convenience sampling method was used to recruit the study participants, who were undergoing definitive RT (excluding those receiving palliative RT) and were available during the study period.

Eighteen head and neck cancer (HNC) patients, consisting of nine men and nine women, aged 20 to 57 years (median age 39.5, CI = 32.3–42.9) were enrolled in this study. The cancers were located in the oral cavity, oropharynx, nasopharynx, larynx, paranasal sinuses, glomus jugulare region, and parotid gland (acinic cell carcinoma). All the patients were non-smokers, and their human papilloma virus (HPV) status was not known. The classification of the primary tumors among the patients was as follows: T1 was observed in 3 patients (16.7%), T2 in 7 patients (38.8%), and T3 in 8 patients (44.4%). Additionally, 50% of the patients presented with N0 nodal status, none were diagnosed with metastasis, and all cases were recorded as M0.

Furthermore, a group of 5 women aged 20 to 42 years (median age 26, CI = 18.0–42.7) with rectal cancer was enrolled. The primary tumors in this group were classified as follows: T1 (33%), T2 (46%), and T3 (21%) stages, with all the patients classified as N0 and M0. All the study participants were exposed to definitive intensity-modulated radiotherapy (IMRT) using 6-MV photons. The HNC patients received a total radiotherapy (RT) dose ranging from 45 Gy to 70 Gy, with an average dose of 64.9 Gy. No surgery or chemotherapy was applied to these patients. The rectal cancer patients were treated with a total RT dose ranging from 45 Gy to 50 Gy, with an average dose of 46 Gy. The patients’ demographics and clinical characteristics are presented in Table 1.

After obtaining ethical approval from the Institutional Review Board (IRB) of Addis Ababa University College of Health Sciences and written informed consent from all the participants, in accordance with the Declaration of Helsinki, approximately 5 mL of venous blood was collected from each participant immediately before and just after the end of radiotherapy (RT). The blood samples were collected using BD Vacutainer SST (Becton, Dickinson and Company, Franklin Lakes, NJ, USA), left to clot for 2 h at room temperature, then centrifuged at 1000× *g* for 10 min. The samples were aliquoted into 1 mL Eppendorf tubes and stored at −80 °C until they were analyzed.

### 2.2. Sample Preparation

The serum samples were thawed and centrifuged at 1000× *g* for 15 min to remove the clots. Then, the samples were treated with the High-Select™ Top14 Abundant Protein Depletion Kit (catalog no. 36370, Thermo Fisher Scientific, Vacaville, CA, USA) according to the manufacturer’s protocol (detailed procedure in Supplementary S1). Initially, 10 µL of the sample were mixed with a resin slurry in a depletion spin column. The blend was end-over-end rotated slowly for one hour at room temperature and then centrifuged for two minutes. The so-obtained filtrate was loaded into a 2 mL Eppendorf vial and lyophilized to a level of about 50 µL to concentrate the protein. The protein concentration was then measured by Bradford assay, and a 20 µg protein sample was prepared based on the determined concentration. For downstream protein analysis, 50 mM ammonium bicarbonate were added to the 20 µg protein sample and incubated at 60 °C for 1 h.

The sample underwent reduction with 4 mM dithiothreitol (DTT) at 95 °C for 30 min and alkylated using 4 mM iodoacetamide (IAA) at room temperature in the dark for 20 min (Sigma Aldrich, Merck KGaA, Darmstadt, Germany). Subsequently, the proteins were trypsin-digested in a 1:50 *w*/*w* ratio (Promega Corporation, Madison, WI, USA) and incubated overnight at 37 °C under gentle shaking. Finally, the samples were purified using Stage Tip C18 and vacuum dried.

### 2.3. Liquid Chromatography–Tandem Mass Spectrometry (LC-MS/MS) Analysis

Around 5 μg of peptides were reconstituted with 0.1% TFA and pumped into a Dionex UltiMate 3000 nano system (Thermo Fisher Scientific, Vacaville, CA, USA) interfaced with an AmaZon ETD mass spectrometer (Bruker Daltonics, Bremen, Germany). The peptide samples were initially loaded on a Pepmap pre-column (2 cm × 100 µm, 5 µm) and then separated using a 25 cm nano column (0.075 µm, Acclaim PepMap100, C18, Thermo Fisher Scientific, CA, USA) at a flow rate of 300 nL/min. Separation was achieved using a multi-step 360 min acetonitrile gradient. A mass spectrometer with nanoBoosterCap-tiveSpray™ ESI source, (ESI Source Solutions, LLC., Woburn, MA, USA) was used in data-dependent acquisition mode.

### 2.4. Mass Spectrometry (MS) Data Analysis

The MS generation utilized enhanced resolution with the trap ICC (ionization and collision control) value set at 400,000, while the ICC target in MS/MS acquisition was raised to 1,000,000. Collision-induced dissociation (CID) was set for fragmentation of the top ten highest intensity MS peaks (top 20) in the MS/MS analysis. The obtained chromatograms were subsequently analyzed with Compass Data Analysis™ v.4.2 (Bruker Daltonics, Bremen, Germany), and the generated mass lists were searched against the UniProt human database using the Mascot search engine (v.2.7.0). The search parameters included trypsin as the enzyme, the likelihood of two missed cleavages, carbamidomethyl (C) as a fixed modification, and oxidation (M) as a variable modification. The mass tolerances were set to 1.2 Da for precursor ions and 0.6 Da for product ions. A global false discovery rate (FDR) of less than 5% was applied to filter the data, and proteins that showed significant differences between the baseline and post-RT were termed as radiation-modulated proteins (RMPs).

### 2.5. Protein Identification and Quantification

Progenesis QI for Proteomics version 4.2 (Non-linear Dynamics, Newcastle, UK) was utilized as the platform for label-free quantification. To summarize, the raw data were imported, and ion intensity maps from all the runs were used in the alignment process, with only alignment scores exceeding 60% being accepted. Peak picking was performed using the default sensitivity settings, with a peak width of 0.15 min and charge states configured to +2, +3, and +4. The survey scan data were used for quantifying peptide ions in the absence of MS/MS data. Data normalization was applied to all the proteins, and identifications were made using Mascot. The protein abundance was determined by summing all the unique peptide normalized ion abundances for each protein across all the runs.

### 2.6. Statistical and Bioinformatics Analysis

IBM SPSS 21 software was employed for statistical analysis. The quantitative data were reported as medians accompanied by their 95% confidence intervals (CI). A 95% CI with a *p*-value < 0.05 was considered statistically significant. To assess the statistical significance for peptides, an ANOVA test was applied, with a *p*-value threshold of ≤0.05 and a fold change ≥ 1.5. Additionally, to address the multiple testing issue, adjusted *p*-values, referred to as q-values, were also calculated, with a threshold of ≤0.01. Proteins with *p* < 0.05 and fold changes ≥1.5 between pre-treatment and post-treatment were classified as radiation-modulated proteins (RMPs).

Pathway and gene ontology (GO) analyses were performed using the SRPLOT R package web server: http://www.bioinformatics.com.cn/SRplot (accessed on 15 October 2024).

## 3. Results

### 3.1. Identification and Quantification of Serum Proteome in Response to Radiotherapy

**Figure 1:**
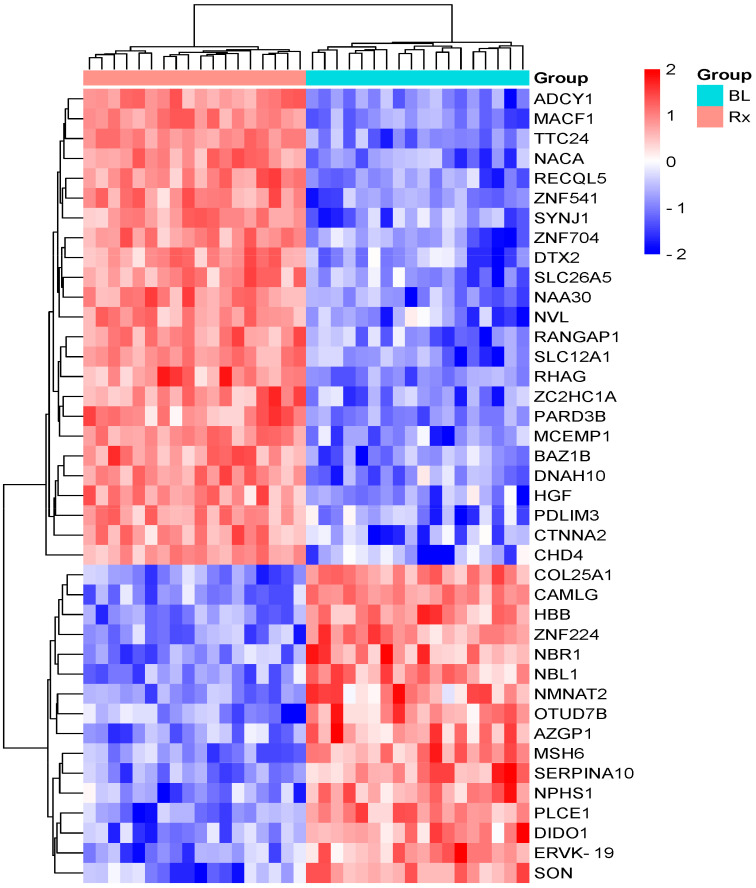
Hierarchical clustering of RMPs between pre-RT and just after the end of RT, with a fold change ≥1.5 and a *p*-value < 0.05 in HNC patients. The rows correspond to the proteins, while the columns represent the patient groups. The relative expression levels are depicted by color intensity, with red indicating high expression and blue signifying low expression. Abbreviations: BL, baseline; Rx, treated.

A shotgun LC-MS/MS technique was utilized for label-free identification and quantification of proteins in serum samples from 18 HNC and 5 rectal cancer patients who underwent radiotherapy (RT), aiming to uncover a molecular profile of alterations caused by ionizing radiation. A total of 830 serum proteins were identified using nano-HPLC-MS/MS (see Supplementary S2), of which forty radiation-modulated proteins (RMPs) were found with a fold change of ≥ 1.5 and a statistically significant ANOVA test *p*-value < 0.05 (Supplementary S3). Of the 40 RMPs, 24 proteins were upregulated, showing increased abundance, while 16 proteins were downregulated, displaying decreased abundance in the post-radiotherapy samples of HNC patients compared to the pre-radiotherapy samples (Figure 1).

Among the 40 RMPs identified, the top five upregulated proteins affected by RT were tyrosine protein kinase BAZ1B (*BAZ1B*), ATP-dependent DNA helicase Q5 (*RECQL5*), zinc finger C2HC domain-containing protein 1A (*ZC2HC1A*), zinc finger protein 541*(ZNF541*), and prestin (*SLC26A5*). The top five downregulated RMPs were OTU domain-containing protein 7B (*OTUD7B*), zinc finger protein 224 (ZNF224), hemoglobin subunit beta (HBB), neuroblastoma suppressor of tumorigenicity 1 (*NBL1*), and nicotinamide/nicotinic acid mononucleotide adenylyltransferase 2 (*NMNAT2*). In patients with rectal cancer, only seven proteins exhibited significant changes in the levels between the samples taken before and immediately after RT (Supplementary S4). Specifically, chromodomain-helicase-DNA-binding protein 4 (*CHD4*), nuclear valosin-containing protein-like (NVL), N-alpha-acetyltransferase 30 (NAA30), microtubule–actin cross-linking factor 1 (isoforms 1/2/3/5) (*MACF1*), and catenin alpha-2 (*CTNNA2*) were found to be upregulated, while DNA mismatch repair protein Msh6 (*MSH6*) and zinc finger protein 224 (*ZNF224*) were downregulated. Even though RT-induced changes can be observed in the serum proteome of the rectal cancer patients, the magnitude of these changes is lower compared to those seen in the HNC patients. The RMPs are listed in Table 2.

**Figure 2:**
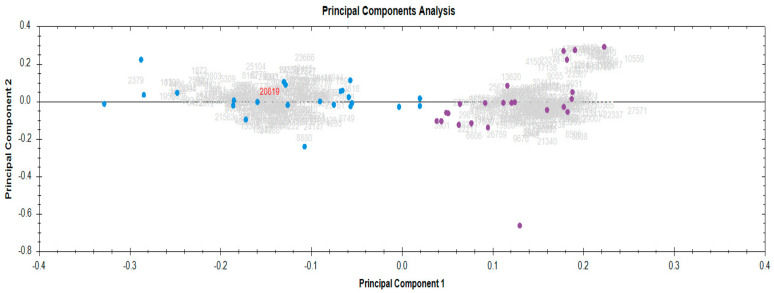
Principal component analysis demonstrating the tight clustering of biological replicates before and after radiotherapy. Baseline, blue; end RT, purple.

A principal component analysis (PCA), based on the unsupervised multivariate analysis of the RMPs, revealed robust experimental consistency in the serum proteomics data. This was supported by the close grouping of biological replicates into two clusters: baseline, blue, and end RT, purple (Figure 2).

### 3.2. Pathway Analysis of DEPs

**Figure 3:**
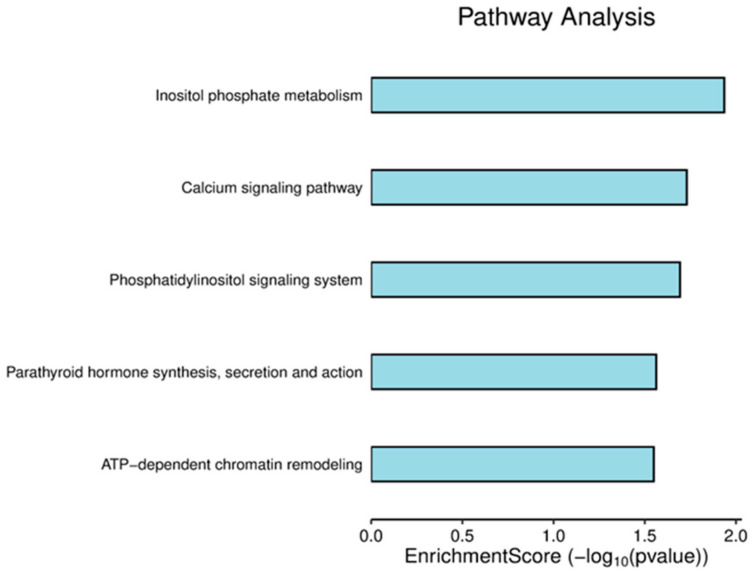
Pathway enrichment score bar plot. The horizontal axis indicates the enrichment score of the RMPs’, while the vertical axis shows the names of the enriched pathways.

A pathway analysis was performed using the SRPLOT R package to identify biological processes affected by radiation, and five pathways were found to be significantly enriched with a *p*-value of less than 0.05 (Figure 3). These pathways include inositol phosphate metabolism; the calcium signaling pathway; the phosphatidylinositol signaling system; parathyroid hormone synthesis, secretion, and action; and ATP-dependent chromatin remodeling (Supplementary S5).

**Figure 4:**
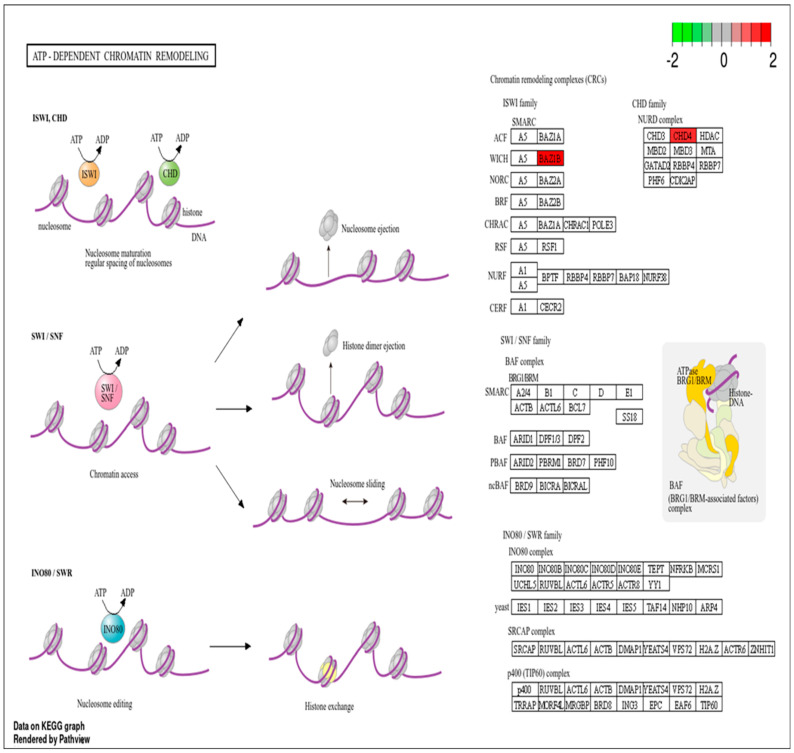
ATP-dependent chromatin remodeling pathway with RMPs. Upregulated RMPs are highlighted in red.

RMPs such as *CHD4* and *BAZ1B* were involved in ATP-dependent chromatin remodeling pathway. Due to the crucial role of this pathway in chromatin remodeling during DNA damage, proteins implicated in this pathway (Figure 4) may serve as a potential signature or clinical biomarker of IR exposure-related DNA damage.

**Figure 5:**
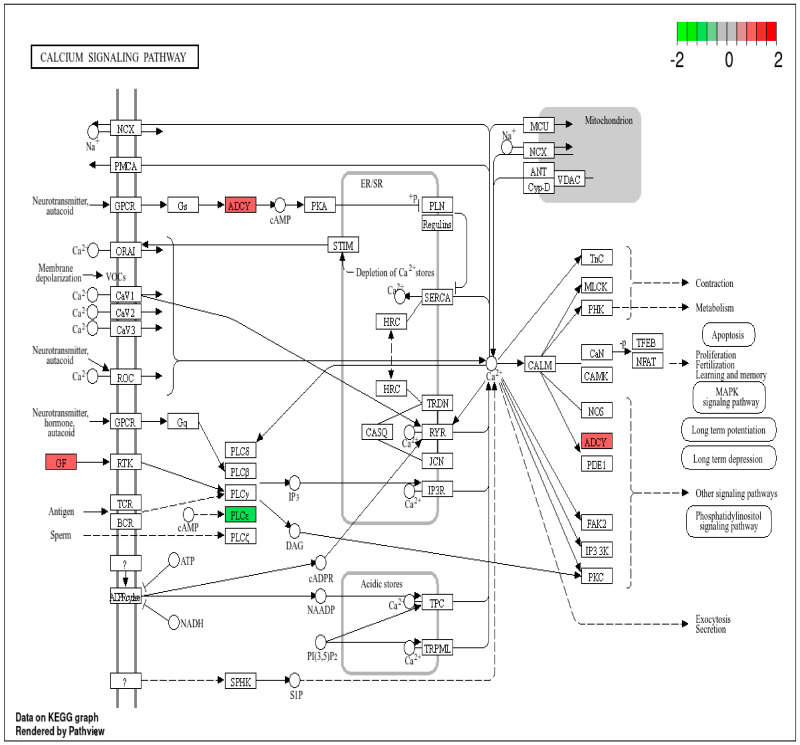
Calcium signaling pathway with RMPs. Upregulated RMPs are highlighted in red, while downregulated RMPs are highlighted in green.

Additionally, RMPs such as *ADCY1* and PL*CE1* are involved in the calcium signaling pathway. Given the role of calcium in cell homeostasis, proteins associated with this pathway (see Figure 5) are likely influenced by radiation exposure and could potentially serve as indicators of IR exposure.

### 3.3. Gene Ontology (GO) Analysis

**Figure 6:**
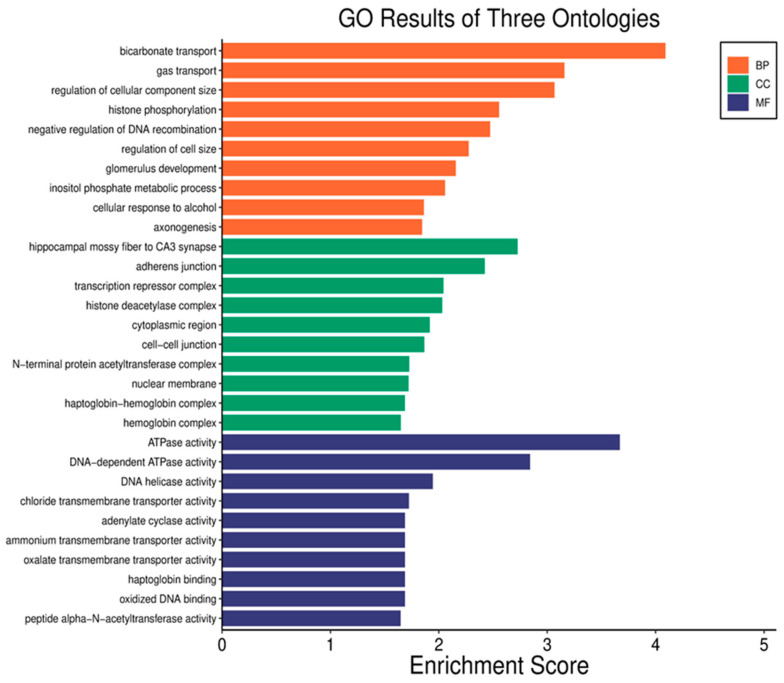
Analysis of gene ontology classification across three categories: biological process (BP), cellular component (CC), and molecular function (MF). The horizontal axis depicts the overrepresentation of RMPs, while the vertical axis shows the names of the ontology classifications.

To explore the molecular processes related to serum proteins influenced by radiation, the genes were annotated using the Gene Ontology (GO) database. The five primary biological processes linked to RMPs affected by radiation included bicarbonate transport, gas transport, regulation of cellular component size, histone phosphorylation, and negative regulation of DNA recombination. The associated cellular components encompassed the hippocampal mossy fiber to CA3 synapse, adherence junctions, transcription complexes, histone deacetylase complexes, and the cytoplasmic region. In terms of molecular functions, the proteins were related to ATPase activity, DNA-dependent ATPase activity, DNA helicase activity, chloride transmembrane transporter activity, and adenylate cyclase activity. Figure 6 illustrates the gene ontology classification analysis of the DEPs.

### 3.4. Ionizing Radiation Clinical Biomarkers

**Figure 7:**
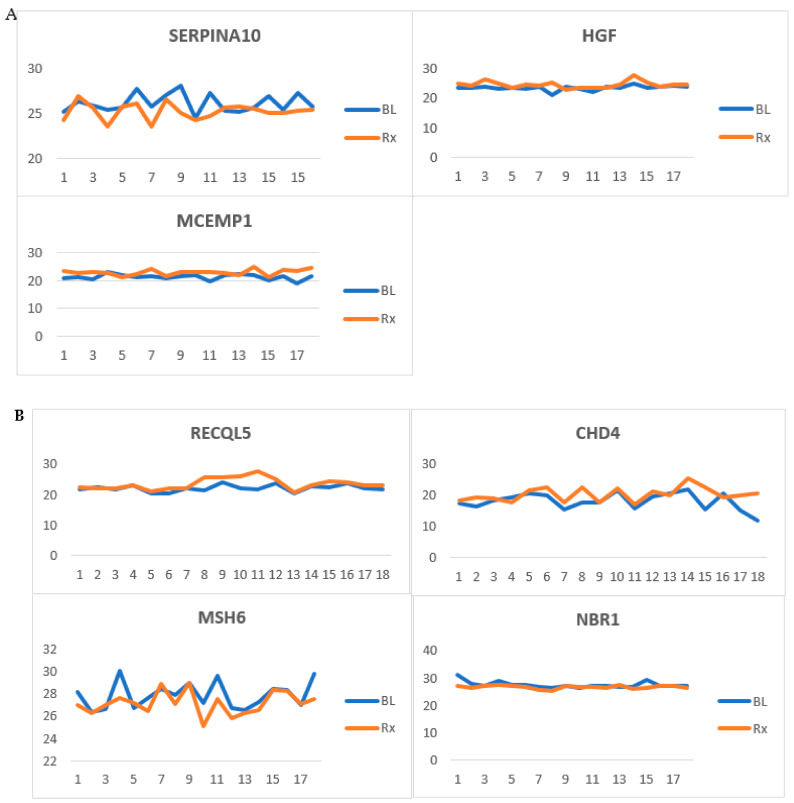

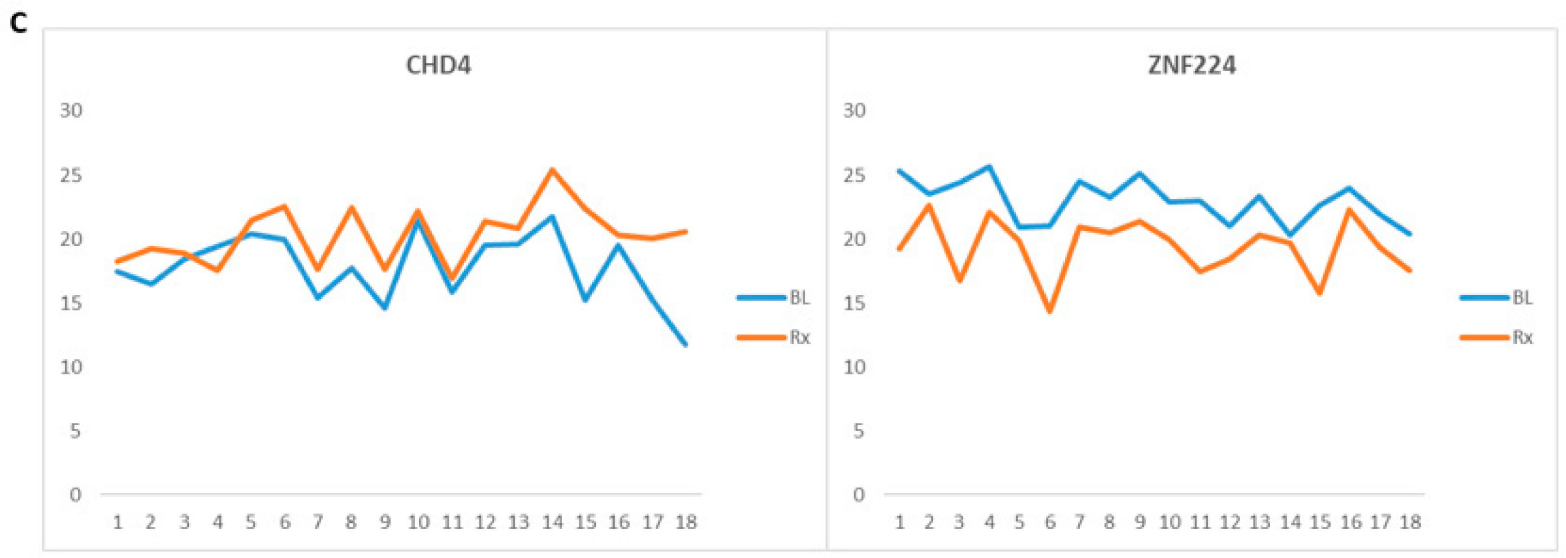
Radiation-modulated proteins (RMPs) whose levels were affected by radiotherapy (RT). (**A**) Proteins involved in acute inflammation and acute phase response in head and neck cancer (HNC) patients. (**B**) RMPs involved in DNA repair and maintenance in HNC patients. (**C**) RMPs involved in DNA repair and maintenance in rectal cancer patients. Samples collected before RT and after RT (*p* < 0.05) are labeled as baseline (BL) and treated (Rx), respectively.

Our study reveals a panel of RMPs affected by RT. We propose a focused subset of serum protein biomarkers specifically targeting those involved in the acute phase response, acute inflammation, immune response, and DNA repair and maintenance. Based on this, we have identified 16 (40%) RMPs related to these processes. Figure 7 illustrates some examples of these RMPs.

## 4. Discussion

Numerous studies have analyzed the effects of ionizing radiation at the proteome level using various models and analytical methods [15]. Using label-free quantitative proteomics, we performed a comprehensive serum proteome analysis and identified a panel of ionizing radiation (IR) biomarkers. A study conducted by Gabriela R et al. identified potential radiation biomarkers in the plasma of 16 leukemia patients who underwent total-body irradiation (TBI). By analyzing blood samples collected before and 24 h after TBI using mass spectrometry, the researchers identified 15 candidate proteins significantly associated with gamma radiation exposure, primarily linked to inflammatory responses and lipid metabolism. Notably, five proteins, namely, C-reactive protein, alpha-amylase 1A, mannose-binding protein C, phospholipid transfer protein, and complement C5, emerged as strong candidates for practical biological dosimetry [16]. This finding aligns with the work of Zebene et al. (2024), who emphasized the importance of proteomic profiling in understanding the cellular response to radiation therapy [17].

In addition to proteomics, transcriptomic and metabolomic approaches are increasingly recognized for their contributions to radiation biodosimetry. Amundson’s article (2022) highlights how transcriptomics has advanced our understanding of gene expression changes following radiation exposure, particularly emphasizing the role of non-coding RNAs and alternative splicing in the radiation response [18]. Similarly, Pannkuk et al. (2017) explored the potential of metabolomics to identify biomarkers associated with biochemical changes resulting from radiation exposure. This approach shows promise for rapid biodosimetry, as it enables the detection of small molecules in biofluids that indicate metabolic changes after radiation exposure. Recent advancements in mass spectrometry have facilitated both untargeted and targeted metabolomic analyses, allowing for the identification of biomarkers and the assessment of overall metabolic dysregulation [19].

Identifying biomarkers for ionizing radiation (IR) in fractionated radiotherapy may assist in developing a preparatory plan for managing medical responses to acute radiation accidents. Fractionated radiotherapy involves delivering a total dose of radiation in smaller fractions over time, allowing for tissue repair and reducing side effects compared to a single high dose [20]. In contrast, acute radiation exposures from accidents involve a high dose delivered rapidly, leading to severe immediate effects, such as acute radiation syndrome (ARS) [21].

As a common mechanism of radiation response, both fractionated RT and acute radiation exposure from accidents trigger similar biological responses, including inflammation and cellular stress. The studies on FDXR and BAX/DDB2 highlight that specific protein biomarkers can be reliably measured after radiation exposure, reflecting the underlying physiological changes irrespective of the exposure context. The study by O’Brien et al. demonstrates that FDXR expression is significantly upregulated in response to various radiation doses, regardless of whether the exposure was from therapeutic or accidental sources. This suggests that FDXR can serve as a universal marker for assessing radiation exposure, making it relevant for both fractionated RT and acute incidents [22].

The findings from Kanagaraj et al. (2024) indicate that BAX and DDB2 are effective biomarkers for acute radiation exposure, displaying persistent upregulation in response to radiation doses. This underscores that similar biomarkers can be utilized to assess acute radiation effects in both clinical (RT) and emergency (accident) settings [23].

Ionizing radiation induces inflammation by damaging cellular structures and triggering the release of reactive species and signaling molecules. In response to this tissue injury and inflammation, acute phase proteins are often upregulated [24,25]. Following exposure to ionizing radiation, the body activates inflammatory pathways to repair the damaged tissues. Additionally, ionizing radiation can affect various proteins involved in cellular responses to damage, apoptosis, and inflammation [26,27].

A study shows that biomarkers, such as C-reactive protein (CRP) and serum amyloid A (SAA), can indicate the inflammatory response due to IR, providing insights into the extent of tissue damage and the body’s healing process [28]. Furthermore, Widlak et al. (2015) observed proteins involved in the acute phase and inflammation, including CRP, SAA4, haptoglobin (HP/HPT), and alpha-1-acid glycoprotein-1 (A1AG1/ORM1), in patients treated with RT [29]. These studies align with our findings, where about 20% of RMPs are involved in acute inflammatory responses, acute phase responses, and immune responses. Key proteins in this biological function include the upregulated adenylate cyclase type 1 (ADCY1), hepatocyte growth factor-like protein (*HGF*), mast cell-expressed membrane protein 1 (MCEMP1), Ran GTPase-activating protein 1 (RANGAP1), zinc-alpha-2-glycoprotein (AZGP*1*), and N-alpha-acetyltransferase 30 (NAA30), along with the downregulated endogenous retrovirus group K member 19 Pro protein (ERVK-19) and protein Z-dependent protease inhibitor (*SERPINA10*).

ADCY1 is significant in signaling pathways activated during inflammation by producing cyclic AMP (cAMP), which modulates various inflammatory processes. The production of cAMP is initiated by the activation of G protein-coupled receptors (GPCRs) that stimulate ADCY to convert ATP to cAMP, thus influencing protein kinase A (PKA) activation and downstream signaling pathways that regulate inflammation [30]. Therefore, *ADCY1* could be considered a signature of ionizing radiation exposure. In a review article conducted by F. Marchetti et al. (2006), a change in the abundance level of a large number proteins was listed, and ADCY1 was one of the identified proteins [31], which is in agreement with our study.

HGF, known for its role in tissue repair and regeneration, can be upregulated following ionizing radiation exposure, aiding in mitigating tissue damage by promoting cell migration and proliferation, while also exhibiting anti-inflammatory properties that help modulate the inflammatory response and reduce oxidative stress in irradiated tissues [32,33,34]. This is in line with our study, where *HGF* was upregulated after RT, suggesting that it could serve as a signature of IR exposure.

In this work, we have observed an upregulation of the MCEMP1 protein. MCEMP1 contributes to the acute inflammatory response by mediating the release of inflammatory mediators, such as histamines and cytokines, essential for initiating and sustaining inflammation, particularly following ionizing radiation exposure [35,36], which makes it a candidate biomarker of IR.

RANGAP1, primarily known for regulating Ran GTPase, which is crucial for the nuclear transport processes, may influence how cells respond to inflammatory signals by regulating the transport of proteins critical for inflammation [37,38]. Given that ionizing radiation can induce inflammatory responses and alter cellular signaling pathways, RANGAP1’s role in protein transport may significantly affect how effectively cells can respond to radiation-induced damage and overall tissue repair mechanisms, thus positioning it as a potential marker of ionizing radiation exposure [39].

AZGP1, involved in metabolism and inflammation, has been linked to cancer progression, with research indicating that its expression can be altered in response to radiotherapy; specifically, AZGP1 is upregulated in radio-resistant colorectal cancer tissues, suggesting a close link to the cellular response to radiation therapy [40,41].

NAA30 (N-alpha-acetyltransferase 30) is an enzyme significant for protein modification through acetylation, which is crucial for regulating gene expression and protein function, influencing how cells respond to stressors, including radiation [42]. Disruption of NAA30 can impair cellular responses to stress, underscoring its importance in protecting cells from damage caused by radiation [43].

ERVK-19 is part of the human endogenous retrovirus K (HERV-K) family, implicated in the regulation of immune responses and influencing the expression of immune-related genes, thus playing a role in the host’s defense mechanisms against infections and diseases [44]. It can affect the transcriptional landscape of the host genome, with its expression upregulated in response to various stimuli, including stress and inflammation, indicating a potential role in the acute phase response and inflammatory processes [45]. Following ionizing radiation exposure, ERVK-19 may influence immune responses, with its expression altered by radiation potentially affecting the immune system’s response to damage [3]. Changes in ERVK-19 expression after radiation exposure can provide insights into the biological effects of radiation and the associated immune response [1], suggesting its potential role as a biomarker for radiation exposure

Lastly, SERPINA10, a component of the serine protease inhibitor (SERPIN) family, is vital for the regulation of inflammation and immune responses. Changes in its expression levels reflect the body’s response to radiotherapy. Alterations in *SERPINA10* expression have been observed in various conditions linked to tissue damage, suggesting its potential as a biomarker for the inflammatory response following radiation exposure [46].

Radiation induces DNA damage, which can lead to significant alterations in chromatin structure and remodeling. When DNA is damaged by radiation, the chromatin structure must be altered to allow access to the damaged sites for repair mechanisms. This involves the remodeling of nucleosomes and changes in histone modifications, which are crucial for facilitating DNA repair [47]. Proteins involved in chromatin-remodeling, such as CHD4 and BAZ1B, were significantly upregulated at the end of RT. *CHD4* is a chromatin-remodeling factor that becomes immobilized on chromatin following ionizing radiation. It coordinates signaling and repair processes, and its depletion can lead to impaired DNA repair and increased DNA breakage [48]. *BAZ1B* is another important protein that helps maintain chromatin’s structure during DNA replication and repair. It interacts with other chromatin-remodeling factors to facilitate access to damaged DNA, ensuring efficient repair processes [25]. Using KEEG pathway analysis, we observed that these proteins (*CHD4* and *BAZ1B*) were involved in the ATP-dependent chromatin remodeling pathway (see Supplementary S3), a pathway that utilizes the energy from ATP hydrolysis to modify chromatin architecture by repositioning, assembling, mobilizing, and restructuring nucleosomes [49]. Therefore, RMPs involved in this pathway may play a significant role in indicating IR exposure.

Radiation can disrupt calcium homeostasis in cells, resulting in increased intracellular calcium levels. Notably, elevations in calcium levels have been observed within seconds to minutes following radiation exposure [26]. RMPs such as ADCY1 and *PLCE1* were involved in the calcium signaling pathway (Supplementary S2), suggesting their role in indicating IR exposure.

As is well known, ionizing radiation (IR) directly affects DNA’s structure, causing breaks, particularly double-strand breaks (DSBs) [50]. DNA damage and repair are key features of how cells respond to ionizing radiation. Therefore, proteins involved in DNA repair and maintenance play a crucial role in addressing radiation-induced DNA damage [12].

In this work, we identified three upregulated proteins (RECQL*5*, CHD4, and TTC2*4*) and five downregulated proteins (ZNF224, MSH6, NBR1, DIDO1, and SON) that are involved in DNA repair and maintenance. Notably, ZNF224, SON, TTC24, and DIDO*1* are implicated in the DNA damage response and repair processes. The expression levels of these proteins can reflect the cellular capacity to manage radiation-induced damage, serving as potential biomarkers for assessing IR exposure, radiosensitivity, and treatment efficacy [3].

Additionally, proteins such as *NBR1* and *MSH6* play critical roles in repairing DNA double-strand breaks and correcting replication errors, respectively, both of which are essential for maintaining genomic stability following radiation exposure [12]. Furthermore, RECQL5 and CHD4 are involved in resolving replication stress and chromatin remodeling, suggesting their potential utility in evaluating the effectiveness of radiotherapy and the extent of cellular recovery from radiation damage [10].

Given their roles in DNA repair and maintenance, these proteins collectively could serve as potential biomarkers of IR exposure. Among the six relevant RMPs identified in rectal cancer patients, two—CHD4 and ZNF224—were specifically found to be involved in DNA repair and maintenance. The smaller number of RMPs in these patients may be attributed to the limited sample size. Supplementary S6 presents the annotated MS/MS spectra of the proteins central to our discussion, which were identified by a single peptide.

Proteins such as zinc finger protein 541 (*ZNF541*), OTU domain-containing protein 7B (*OTUD7B)*, solute carrier family 12 member 1 (SLC12A1), synaptojanin-1 (SYNJ1), nascent polypeptide-associated complex subunit alpha (*NACA*), and nuclear valosin-containing protein-like (NVL) are not classified as definitive biomarkers for ionizing radiation. However, they are involved in stress response, DNA repair, and cellular signaling, suggesting that they could contribute to a radiation response signature.

ZNF541 may play a role in transcriptional regulation and cellular stress responses (GeneCards, 2023) [51].

OTUD7B is a deubiquitinating enzyme that regulates protein stability and is involved in the cellular response to stress. It has been shown to play a role in DNA damage response pathways, which are critical for maintaining genomic integrity under stress conditions [52].

SLC12A1, a solute carrier protein, is primarily known for its role in ion transport. Changes in ion homeostasis can affect cellular signaling pathways and stress responses, particularly in the context of oxidative stress and DNA damage [53]. SYNJ1 is involved in endocytosis, synaptic function, and maintaining cellular homeostasis [54]. Its role in maintaining cellular homeostasis suggests it may contribute to the cellular response to DNA damage.

NACA is a chaperone that plays an important role in the synthesis of nascent polypeptides by folding them correctly into proteins, thereby preventing their misfolding. In conditions of stress, for example, ionizing radiation, its role becomes critical in managing the increased load of protein misfolding. Indeed, *NACA* mediates proper folding of cellular proteins, thereby granting resistance and integrity to the cell against such stressors [55,56].

NVL takes part in RNA processing directly, including splicing and regulation of RNA stability, which is very important for the normal expression of genes and cellular health in general. Given its involvement in ribonucleic acid (RNA) metabolism, *NVL* could also play a role in the response to radiation-induced stress at the cellular level. Cells exposed to ionizing radiation show damage regarding the integrity and function of RNA, and NVL might have a modulatory role in maintaining proper RNA processing [57,58,59].

In general, we can conclude that the upregulation and downregulation of RMPs associated with acute phase and inflammatory responses, as well as DNA repair and maintenance, may represent a general serum proteome signature in response to IR exposure.

Other proteins affected by RT include CTNNA2, SLC26A5, DTX2, NPHS1, CAMLG, DNAH10, PDLIM3, HBB, PARD3B, ZNF704, NBL1, COL25A1, NMNAT2, RHAG, and ZC2HC1A. Although not established biomarkers of IR exposure, their significantly altered expression levels after RT suggest they may play a role in the radiation response and could serve as potential indicators of IR exposure in future studies.

On the other hand, it is important to consider other genotoxic agents that can damage genetic material within a cell. A study by Carina Ladeira and Lenka Smajdova highlights the significance of genotoxicity biomarkers beyond ionizing radiation (IR), linking exposure to various harmful substances with disease development, particularly cancer. The study identifies key genotoxic agents, including air pollutants, heavy metals, pesticides, food additives, and contaminants, as well as mobile phone radiation and formaldehyde [60].

In our research, we identified several proteins modulated following radiation therapy (RT), indicating a dynamic response of cellular pathways to IR and suggesting potential biomarkers for monitoring its effects. However, while these proteins show significant modulation post-RT, establishing their specificity to ionizing radiation, as opposed to other genotoxic agents, remains a challenge that warrants further research.

## 5. Conclusions

This study has identified a panel of serum proteins that are significantly modulated in response to ionizing radiation (IR) exposure in patients undergoing radiotherapy (RT). By employing a quantitative proteomic approach, we have uncovered a comprehensive molecular signature associated with IR-induced cellular responses. A significant number (approximately 40%) of the identified radiation-modulated proteins (RMPs) were implicated in crucial biological processes, including acute phase response, DNA repair, and inflammation. The serum proteome signature proposed in this study has the potential to serve as an IR biomarker for early detection and effective clinical management of IR-related injuries. Ultimately, while our findings provide a foundation for understanding protein modulation following RT, the nuanced interplay between radiation exposure and other genotoxic influences necessitates further exploration to confirm the specificity of these biomarkers. Future research should focus on validating these biomarkers in larger cohorts and exploring their utility in various clinical settings to improve patient outcomes in radiation therapy.

## Figures and Tables

**Table 1:** Patients’ demographics and clinical characteristics (N = 23).

Clinical and Demographic Features	HNC = 18	*p*-Value
Median age (95% CI range)	39.5 (32.3–42.9)	
Radiation dose (95% CI)	70 (60.8–69.1)	
Gender		
Male	9 (50%)	0.00
Female	9 (50%)	
T1–T2 (T1 = 3, T2 = 7)	10 (55.5)	0.00
T3 = 8	8 (44.4%)	
N0	9 (50%)	0.00
N+	9 (50%)	
Clinical and demographic features	Rectal = 5	
Median age (95% CI range)	26 (18.0–42.7)	
Radiation dose (95% CI)	45 (43–49)	
Gender		
Male	0 (0.0%)	
Female	5 (100%)	
TNM		
T1–T2 (T1 = 2, T2 = 2)	4 (80%)	0.082
T3 = 8 (T3 = 1)	1 (20%)	
N0	5 (100%)	

**Table 2:** Radiation-modulated proteins (RMPs) before and after treatment in HNC patients.

Protein Description	Gene Name	Acc. UniProtKB	Fold Change (Tx/CTR)	*p*-Value	Regulation
Tyrosine protein kinase BAZ1B	BAZ1B	Q9UIG0	10.5	0.02	Upregulated
ATP-dependent DNA helicase Q5	RECQL5	O94762	5.2	0.04	Upregulated
Zinc finger C2HC domain-containing protein 1A	ZC2HC1A	Q96GY0	5.2	0.04	Upregulated
Zinc finger protein 541	ZNF541	Q9H0D2	3.7	0.02	Upregulated
Prestin	SLC26A5	P58743	3.2	0.02	Upregulated
Ran GTPase-activating protein 1	RANGAP1	P46060	3.2	0.01	Upregulated
Solute carrier family 12 member 1	SLC12A1	Q13621	3.1	0.04	Upregulated
Dynein heavy chain 10, axonemal	DNAH10	Q8IVF4	3.0	0.03	Upregulated
Catenin alpha-2	CTNNA2	P26232	2.9	0.02	Upregulated *
*Chromodomain-helicase-DNA-binding protein 4	CHD4	Q14839	2.6	0.03	Upregulated *
Synaptojanin-1	SYNJ1	O43426	2.3	0.03	Upregulated
Ammonium transporter Rh type A	RHAG	Q02094	2.3	0.04	Upregulated
Hepatocyte growth factor-like protein	HGF	P14210	2.2	0.04	Upregulated
Adenylate cyclase type 1	ADCY1	Q08828	2.1	0.01	Upregulated
PDZ and LIM domain protein 3	PDLIM3	Q53GG5	2.1	0.03	Upregulated
Mast cell-expressed membrane protein 1	MCEMP1	Q8IX19	1.8	0.03	Upregulated
N-alpha-acetyltransferase 30	NAA30	Q147X3	1.8	0.03	Upregulated *
Zinc finger protein 704	ZNF704	Q6ZNC4	1.7	0.04	Upregulated
Tetratricopeptide repeat protein 24	TTC24	A2A3L6	1.6	0.01	Upregulated
Nascent polypeptide-associated complex subunit alpha, muscle-specific form	NACA	E9PAV3	1.6	0.03	Upregulated
Probable E3 ubiquitin protein ligase DTX2	DTX2	Q86UW9	1.6	0.02	Upregulated
Partitioning defective 3 homolog B	PARD3B	Q8TEW8	1.5	0.03	Upregulated
Microtubule–actin cross-linking factor 1, isoforms 1/2/3/5	MACF1	O94854	1.5	0.02	Upregulated *
Nuclear valosin-containing protein-like	NVL	O15381	1.5	0.02	Upregulated *
DNA mismatch repair protein Msh6	MSH6	P52701	0.5	0.04	Downregulated *
Collagen alpha-1(XXV) chain	COL25A1	Q9BXS0	0.5	0.04	Downregulated
Protein SON OS = Homo sapiens	SON	P18583	0.5	0.02	Downregulated
Protein Z-dependent protease inhibitor	SERPINA10	Q9UK55	0.5	0.01	Downregulated
1-phosphatidylinositol 4,5-bisphosphate phosphodiesterase epsilon-1	PLCE1	Q9P212	0.49	0.04	Downregulated
Death-inducer obliterator 1	DIDO1	Q9BTC0	0.49	0.007	Downregulated
Endogenous retrovirus group K member 19 Pro protein	ERVK-19	O71037	0.48	0.02	Downregulated
Zinc-alpha-2-glycoprotein	AZGP1	P25311	0.47	0.003	Downregulated
Nephrin OS = Homo sapiens	NPHS1	O60500	0.47	0.02	Downregulated
Next to BRCA1 gene 1 protein	NBR1	Q14596	0.39	0.02	downregulated
Calcium signal-modulating cyclophilin ligand	CAMLG	P49069	0.38	0.02	Downregulated
Nicotinamide/nicotinic acid mononucleotide adenylyltransferase 2	NMNAT2	Q9BZQ4	0.36	0.04	Downregulated
Neuroblastoma suppressor of tumorigenicity 1	NBL1	P41271	0.32	0.04	Downregulated
Hemoglobin subunit beta	HBB	P68871	0.32	0.03	Downregulated
Zinc finger protein 224	ZNF224	Q9NZL3	0.19	0.01	Downregulated *
OTU domain-containing protein 7B	OTUD7B	Q6GQQ9	0.15	0.03	Downregulated

* RMPs significantly identified in both data sets (HNC and rectal cancer).

## Data Availability

All the data generated or analyzed during this study are included in this manuscript. In addition, the primary data can be obtained on https://doi.org/10.5281/zenodo.14620172.

## Supplementary Materials

The following supporting information can be downloaded at: https://www.mdpi.com/article/10.3390/cancers17061010/s1, Supplementary S1: Procedure; Supplementary S2: Total identified proteins-HNC; Supplementary S3: Statistically significant proteins-HNC; Supplementary S4: Statistically significant proteins-Rectal; Supplementary S5: Pathway Analysis; Supplementary S6: MS/MS-spectrum.

## Abbreviations

ARS	Acute radiation syndrome
BD-SST	Blood collection serum-separator
RT	Radiotherapy
BP	Biological process
CC	Cellular component
DNA	Deoxyribonucleic acid
DSBs	Double-strand breaks
FC	Fold change
GLOBOCAN	Global Cancer Observatory
GO	Gene ontology
GY	Gray
HNC	Head and neck cancer
HPLC	High performance liquid chromatography
IARC	International Agency for Cancer Research
IMRT	Intensity-modulated radiotherapy
IPA	Ingenuity pathway analysis
IR	Ionizing radiation
MF	Molecular function
MS	Mass spectrometer
PCA	Principal component analysis
PPI	Protein interaction
RMPs	Radiation-modulated proteins
RNA	Ribonucleic acid
TFA	Trifluoroacetic acid
TNM	Tumor, nodes, and metastasis
