# Supplementary material for: Serum-Based Proteomic Approach to Identify Clinical Biomarkers of Radiation Exposure
Source: Cancers (Basel). 2025 Mar 17;17(6):1010. doi: 10.3390/cancers17061010 (PMC11940482; doi:10.3390/cancers17061010)
Supplement: Supplementary file 1 [file cancers-17-01010-s001.zip › Supplementary S1.pdf]

Procedure

High-Select™ Top14 Abundant Protein Depletion Mini Spin Columns Kit (Thermo Fisher Scientific,USA).

Sample preparation

The serum samples were thawed and subjected to pretreatment using the High-Select™ Top14 Abundant Protein Depletion Mini Spin Columns Kit (Thermo Fisher Scientific,USA) according to the manufacturer's protocol. This kit is designed to remove the 14 most abundant proteins present in the serum, including Albumin, IgA, IgD, IgE, IgG, IgG (light chains), IgM, Alpha-1-acid glycoprotein, Alpha-1-antitrypsin, Alpha-2-macroglobulin, apolipoprotein A1, fibrinogen, haptoglobin, and transferrin.

Procedure to Remove high-abundance top 14 proteins in mini format

1. Equilibrate the depletion spin column to room temperature.

2. Remove the column screw cap and add up to 10 µL of sample directly to the resin slurry in the column.

3. Cap the column and invert the column several times until the resin is completely homogenous in solution.

4. Incubate the mixture in the column with gentle end-over-end mixing for 10 minutes at room temperature. Make sure the sample mixes with the resin during incubation period. Alternatively, gently vortex every few minutes.

5. After incubation, snap off the bottom closure and loosen the top cap. Place the mini column into a 2 mL collection tube and centrifuge at 1,000 × g for 2 minutes.

6. Discard the column containing the resin.

7. Filtrate contains sample with albumin, IgG, and other abundant proteins removed. Use for further processing or store at -20°C for later use.

The depleted sample will be in 10 mM PBS and 0.02% sodium azide, pH 7.4.

Note: Sample processing will depend on the type of downstream analysis and may require buffer exchange, lipids and other metabolite removal and/or concentration for 2D gel electrophoresis and

MS analysis. Use Thermo Scientific™ Pierce™ Protein Concentrators for buffer exchanging and/or concentrating
