# Supplementary material for: Serum-Based Proteomic Approach to Identify Clinical Biomarkers of Radiation Exposure
Source: Cancers (Basel). 2025 Mar 17;17(6):1010. doi: 10.3390/cancers17061010 (PMC11940482; doi:10.3390/cancers17061010)

MS/MS-Spectrum

Hepatocyte Growth Factor-like Protein (HGF)

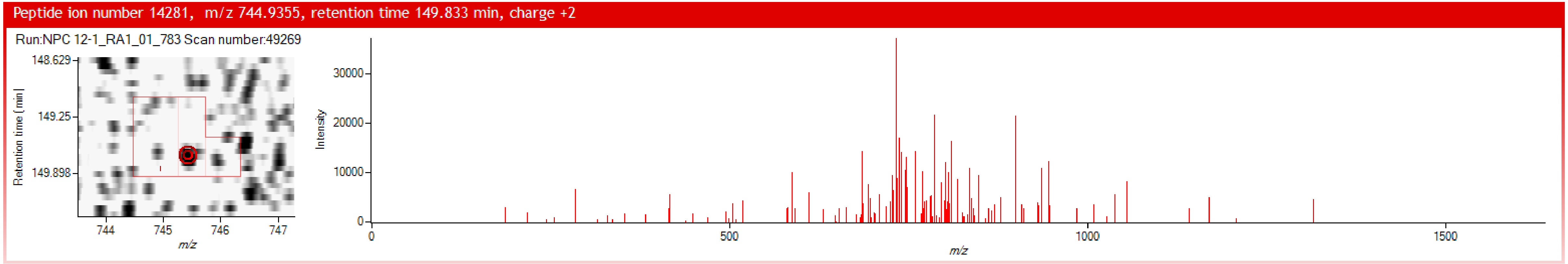

Mast Cell-expressed Membrane Protein 1 (MCEMP1)

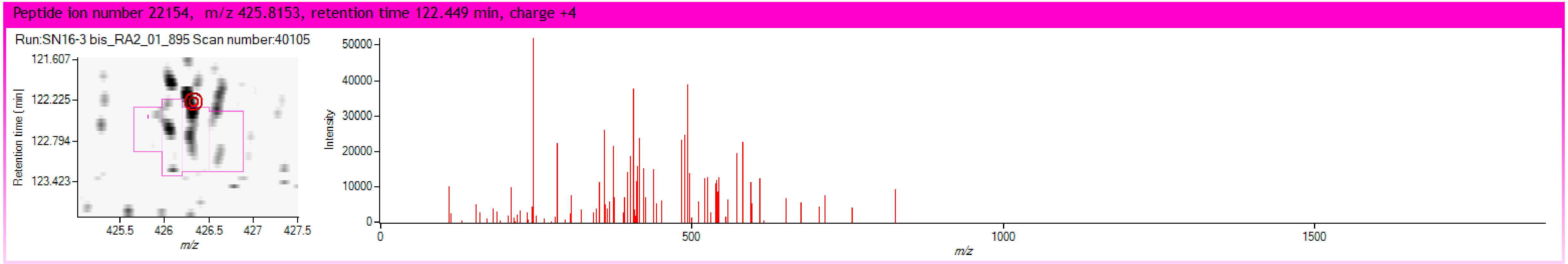

N-alpha-acetyltransferase 30 (NAA30)

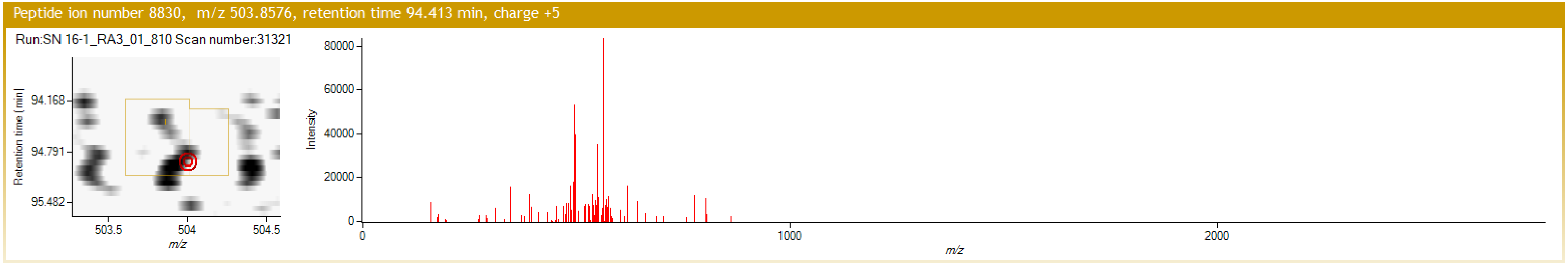

Protein Z-dependent Protease Inhibitor (SERPINA10)

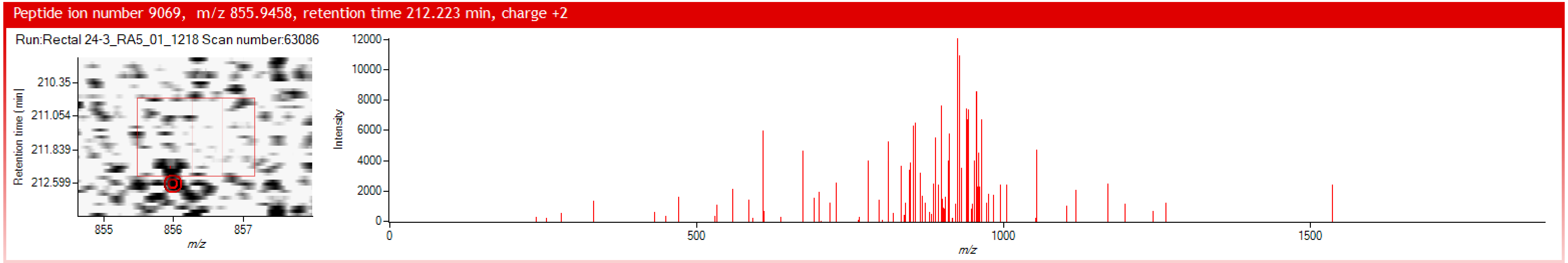

TTC24

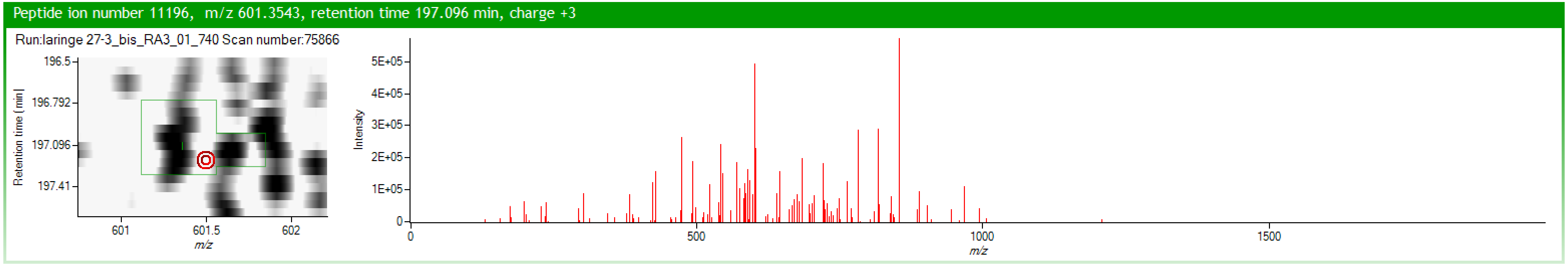

ZNF224

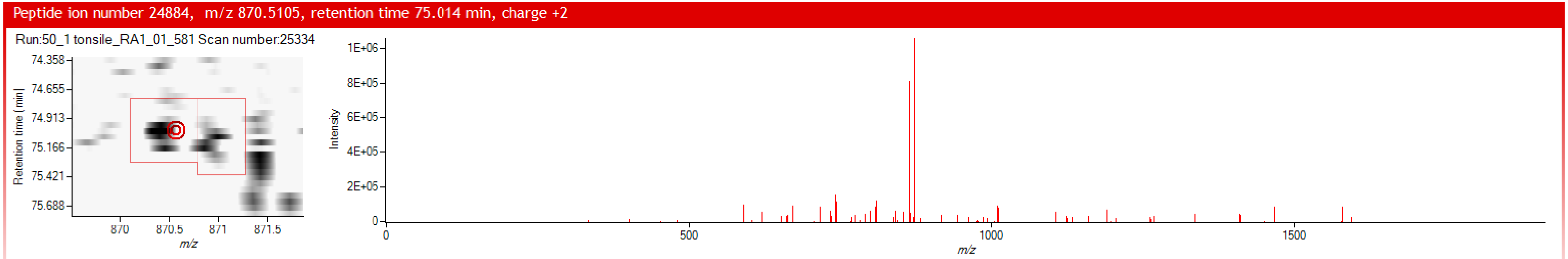

NBR1

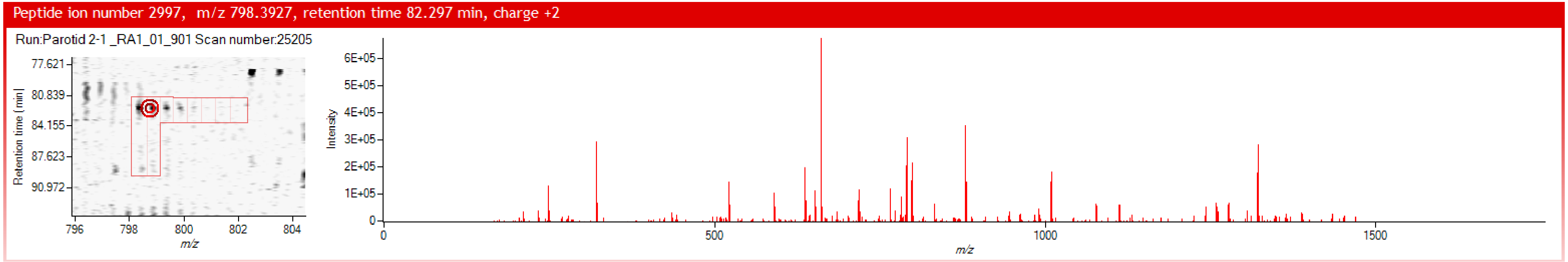

SCL12A1

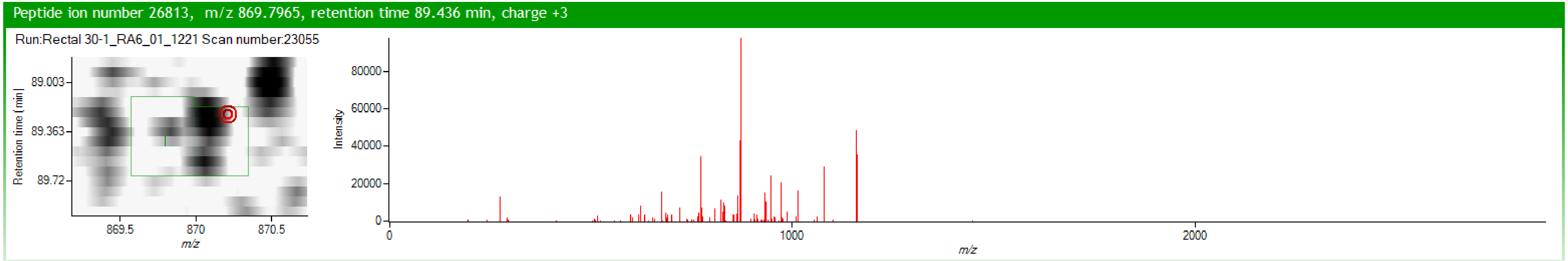

Supplement: Supplementary file 1 [file cancers-17-01010-s001.zip › Supplementary S6.pdf]
